# Use of Cardiovascular Drugs for Primary and Secondary Prevention of Cardiovascular Disease Among Rural-Dwelling Older Chinese Adults

**DOI:** 10.3389/fphar.2020.608136

**Published:** 2020-12-18

**Authors:** Lin Cong, Yifei Ren, Tingting Hou, Xiaolei Han, Yi Dong, Yongxiang Wang, Qinghua Zhang, Rui Liu, Shan Xu, Lidan Wang, Yifeng Du, Chengxuan Qiu

**Affiliations:** ^1^ Department of Neurology, Shandong Provincial Hospital, Cheeloo College of Medicine, Shandong University, Jinan, China; ^2^ Department of Neurology, Shandong Provincial Hospital Affiliated to Shandong First Medical University, Jinan, China; ^3^ Shandong Provincial Clinical Research Center for Neurological Diseases, Jinan, China; ^4^ Aging Research Center and Center for Alzheimer Research, Department of Neurobiology, Care Sciences and Society, Karolinska Institutet and Stockholm University, Stockholm, Sweden

**Keywords:** cardiovascular drugs, cardiovascular disease, prevalence, prevention, rural

## Abstract

Cardiovascular risk factors and related disorders are common among older adults, and use of various classes of cardiovascular (CV) drugs could reduce the risk of cardiovascular disease (CVD). However, data are sparse with regard to the use of CV drugs among rural-dwelling older adults in China. Therefore, this population-based study aimed to describe use of CV drugs among older adults living in the rural communities in China, while taking into account the use of CV drugs for primary and secondary prevention of CVDs. This study included 5,246 participants (age ≥65 years; 57.17% women; 40.68% illiteracy) in the baseline examination of the MIND-China study. In March-September 2018, data on health-related factors, CVDs (ischemic heart disease, atrial fibrillation, heart failure, and stroke), and CV drug use were collected via face-to-face survey, clinical examination, and laboratory tests. We classified CV drugs according to the Anatomical Therapeutic Chemical classification system for western medications and specific cardiovascular effects for the products of traditional Chinese medicine (TCM). We conducted descriptive analysis. The overall prevalence of major cardiovascular risk factors ranged from 14.30% in diabetes and 23.81% in dyslipidemia to 66.70% in hypertension, and CVDs affected 35.07% of all participants (36.28% in women vs. 33.47% in men, *p* = 0.035). In the total sample, calcium channel blockers (C08) were most commonly used (10.39%), followed by TCM products (7.64%), hypoglycemic agents (A10, 4.73%), renin-angiotensin system (RAS)-acting agents (C09, 4.61%), and lipid-lowering agents (C10, 4.17%). The proportions of CV drugs for primary prevention (i.e., use of CV drugs among people without CVD) were 3.14% for antithrombotic agents (mainly aspirin), 1.38% for lipid-lowering agents, and 3.11% for RAS-acting agents; the corresponding figures for secondary prevention (i.e., use of CV drugs among people with CVD) were 13.97%, 9.35%, and 7.39%. In conclusion, despite highly prevalent cardiovascular risk factors and CVDs, a fairly low proportion of the rural-dwelling older adults take CV medications for primary and secondary prevention. Notably, TCM products are among the most commonly used CV drugs. These results call for additional efforts to promote implementation of the evidence-based recommendations for prevention of CVDs in the primary care settings.

## Introduction

Cardiovascular disease (CVD) is the leading cause of premature death in China, contributing to ∼40% of all deaths (Zhou et al., 2016). Since the 1990s, the age-standardized mortality of CVD has steadily declined among urban residents in China, but the declining trend was not evident among people living in the rural areas (Sun et al., 2017).

Cardiovascular (CV) drugs are the most commonly used therapeutic classes of drugs in older adults (Schwartz et al., 2019). It has been well established that various CV drug therapies among people at risk could reduce the risk of developing CVD and death from CVD (Khatib et al., 2016). CV drugs, such as renin-angiotensin system (RAS)-acting agents, beta-blockers, antithrombotic agents, and lipid-lowering drugs, have been widely recommended by the international guidelines for the primary or secondary prevention of CVD (Smith et al., 2011; Arnett et al., 2019).

Despite the strong scientific evidence, a substantial gap between the guidelines of CV drug therapies and the implementation in primary health care remained in China (Du et al., 2019). The main risk factors for CVD, such as hypertension, dyslipidemia, and diabetes mellitus, remained highly prevalent and poorly managed in China, especially among elderly residents living in the rural areas (Song et al., 2014). Data from both the Prospective Urban Rural Epidemiological (PURE)-China study (Yusuf et al., 2011) and the China Kadoorie Biobank (CKB) study (Chen et al., 2014) showed that less than one-third of people with CVDs received the proven CV drugs for secondary prevention.

The majority of cardiovascular epidemiological studies in China have focused on urban populations, and relatively few studies on the management of CVDs in rural settings are available. Studying preventive and therapeutic CV drug use in the rural areas in China is important because over 50% of people live outside cities, and optimal use of scarce healthcare resources is vital (Liu et al., 2008). Therefore, the objective of this population-based study was to determine the prevalence of CV drug use among rural-dwelling older Chinese adults (age ≥65 years) and to identify CV drugs commonly used for primary and secondary prevention of CVDs.

## Methods

### Study Design and Participants

This population-based study is planned within the Multimodal Interventions to Delay Dementia and Disability in Rural China (MIND-China) study (Kivipelto et al., 2020). In brief, the MIND-China study targeted residents from 52 villages in the rural areas of Western Shandong Province (Yanlou Town of Yanggu county). In March-September 2018, baseline assessments and screenings for participants were completed, during which 5,765 subjects who were 60 years of age or older were examined. After exclusion of subjects aged 60–64 years (n = 519) due to relatively low participating rate, a total number of 5,246 (91.0%) people were included in this analysis.

The MIND-China protocol has been approved by the Ethics Committee at Shandong Provincial Hospital, Jinan, Shandong, China. Written informed consent was obtained from all participants, or in the case of cognitively impaired persons, from a proxy (usually a guardian or a family member).

### Data Collection and Definitions

The baseline examination included a face-to-face interview carried out by trained research staff with a structured questionnaire (e.g., lifestyle and medical history), physical and neurological examination (e.g., height, weight, blood pressure, pulse rate, and neurological disorders), electrocardiogram (ECG) examination, and laboratory test (e.g., fasting blood glucose and lipids). During the interview, a detailed medical history was sought from participants with the questions: “Has a doctor EVER told you that you had the following disease?”, followed by a list of major health conditions, such as hypertension, dyslipidemia, diabetes mellitus, ischemic heart disease (IHD), heart failure (HF), atrial fibrillation (AF), and stroke. Then participants were asked: “Are you currently (in the past two weeks) taking any medicines, tablets or injections of any kind drugs, either you buy yourself or are prescribed by your doctor?”. If the answer was ‘Yes’, details of medicines were recorded, including name, dosage, and frequency. All western medications were classified and coded according to the Anatomical Therapeutic Chemical (ATC) classification system. The classes of CV drugs included antithrombotic agents (ATC code B01), diuretics (C03), beta-blockers (C07), calcium channel blockers (C08), renin-angiotensin system (RAS)-acting agents (C09), lipid-lowering drugs (C10), and cardiac drugs (C01). Because products of traditional Chinese medicine (TCM) were widely provided to the local residents by the primary healthcare institutions, we also recorded TCM products that were used for the treatment and control of CVDs and risk factors, and classified the TCM products according to their specific cardiovascular effects (e.g., TCM products for CVDs, hypertension, lipids lowering, and diabetes). Numbers of concurrent use of CV drugs included both western medications and TCM products.

Hypertension was defined as systolic blood pressure of at least 140 mmHg or diastolic blood pressure of at least 90 mmHg, or use of any antihypertensive drugs (ATC codes C02, C03, C07-C09, and TCM products for lowering blood pressure) in the past two weeks (James et al., 2014; Joint Committee for Guideline Revision, 2019). Dyslipidemia was defined as total cholesterol (TC) ≥240 mg/dl, low-density lipoprotein cholesterol (LDL-C) ≥160 mg/dl, high-density lipoprotein cholesterol (HDL-C) <40 mg/dl, triglycerides (TG) ≥200 mg/dl, or use of lipid-lowering medications (ATC code C10 and TCM products for lowering lipids) (Wang et al., 2020). Diabetes mellitus was defined as having a fasting blood glucose ≥126 mg/dl or self-reported physician diagnosis of diabetes or use of diabetes medications (ATC code A10 and TCM products for lowering blood glucose) (American Diabetes Association, 2019). Obesity for Chinese adults was defined as body mass index (BMI) ≥28 kg/m^2^ (Wan et al., 2017). IHD was identified according to self-report history of myocardial infarction (MI), angina, coronary intervention, or pathological Q waves on ECG; clinical stroke as a combination of self-reporting history of stroke or the judgment of clinical stroke by a neurologist or physician via neurological examination; and HF as self-reported physician diagnosis of HF. The diagnosis of AF was made based on ECG examination. CVDs included IHD, HF, AF, and stroke (Du et al., 2019; Liu et al., 2019).

Drug use for primary prevention of CVD was defined as use of CV drugs among persons who were free from CVDs, and drug use for secondary prevention as use of CV drugs among people who had CVDs.

### Statistical Analysis

We performed descriptive analysis on the use of CV drugs. We reported frequency (%) for categorical variables (e.g., disease and medication intakes), and mean (SD) for continuous variables. We compared the medication use between genders and different CVDs by using chi-square tests. All statistical analyses were performed by using SAS 9.4 software (SAS Institute Inc., 2013; Cary, NC, United States).

## Results

### Characteristics of the Study Population

Table 1 details the demographic features of the study population. The average age of all participants was 71.74 years (SD 5.52), 57.17% were women, and 40.68% were illiterate. Out of the 5,246 participants, hypertension was diagnosed in 3,499 (66.70%) persons, diabetes in 750 (14.30%), and dyslipidemia in 1,249 (23.81%). Overall, 1840 (35.07%) participants were ascertained to have CVD, including 1,152 (21.96%) with IHD, 153 (2.92%) with HF, 84 (1.60%) with AF, and 840 (16.03%) with clinical stroke. The crude prevalence of hypertension, diabetes, dyslipidemia, and IHD was higher in women than in men (*p* < 0.05), whereas the prevalence of stroke was higher in men than in women (17.40% vs. 14.97%, *p* = 0.018). As expected, compared with participants who were free from CVD, people with CVD had a higher prevalence of hypertension (63.48% vs. 72.66%, *p* < 0.05), diabetes (11.54% vs. 19.40%, *p* < 0.05), and dyslipidemia (20.35% vs. 30.22%, *p* < 0.05).

**TABLE 1:** Characteristics of study participants in the total sample and by gender and cardiovascular disease.

Characteristics	Total Sample (n = 5,246)	Gender			Cardiovascular disease		
		Men (n = 2,247)	Women (n = 2,999)	*p*-value^a^	No (n = 3,406)	Yes (n = 1840)	*p*-value^a^
Age (years), mean (SD)	71.74 (5.52)	71.58 (5.36)	71.86 (5.63)	0.071	71.51 (5.49)	72.16 (5.54)	<0.0001
Age group (years)				0.001			<0.0001
65–69	2,209 (42.11)	969 (43.12)	1,240 (41.35)		1,535 (45.07)	674 (36.63)	
70–74	1,688 (32.18)	703 (31.29)	985 (32.84)		1,040 (30.53)	648 (35.22)	
75–79	792 (15.10)	371 (16.51)	421 (14.04)		485 (14.24)	307 (16.68)	
≥80	557 (10.62)	204 (9.08)	353 (11.77)		346 (10.16)	211 (11.47)	
Education level				<0.0001			0.741
Illiteracy	2,134 (40.68)	325 (14.46)	1809 (60.32)		1,388 (40.75)	746 (40.54)	
Primary school	2,270 (43.27)	1,210 (53.85)	1,060 (35.35)		1,481 (43.48)	789 (42.88)	
Middle school and above	842 (16.05)	712 (31.69)	130 (4.33)		537 (15.77)	305 (16.58)	
Obesity	1,201 (22.89)	427 (19.00)	774 (25.81)	<0.0001	682 (20.02)	519 (28.21)	<0.0001
Current smoking	1,064 (20.28)	1,025 (45.62)	39 (1.30)	<0.0001	762 (22.37)	302 (16.41)	<0.0001
Current drinking	1931 (36.81)	1713 (76.23)	218 (7.27)	<0.0001	1,301 (38.20)	630 (34.24)	0.005
Hypertension	3,499 (66.70)	1,444 (64.26)	2055 (68.52)	0.001	2,162 (63.48)	1,337 (72.66)	<0.0001
Diabetes	750 (14.30)	255 (11.35)	495 (16.51)	<0.0001	393 (11.54)	357 (19.40)	<0.0001
Dyslipidemia	1,249 (23.81)	352 (15.67)	897 (29.91)	<0.0001	693 (20.35)	556 (30.22)	<0.0001
Cardiovascular disease	1840 (35.07)	752 (33.47)	1,088 (36.28)	0.035	—	—	—
Ischemic heart disease	1,152 (21.96)	409 (18.20)	743 (24.77)	<0.0001	—	—	—
Stroke	840 (16.03)	391 (17.40)	449 (14.99)	0.018	—	—	—
Heart failure	153 (2.92)	60 (2.67)	93 (3.10)	0.359	—	—	—
Atrial fibrillation	84 (1.60)	39 (1.74)	45 (1.50)	0.502	—	—	—

Note: Data are n (%), unless otherwise specified.

a p-value is for the test of differences between women and men or between participants without and with cardiovascular disease.

### Cardiovascular Drug Use in the Total Sample

Overall, calcium channel blockers (C08) were most commonly used (10.39%), followed by TCM products (7.64%) and antithrombotic agents (B01, 6.94%). The overall prevalence of using other CV drugs was less than 5%, ranging from around 1.5% for diuretics (C03), beta-blockers (C07), and cardiac therapy (C01) to around 4.5% for lipid-lowering agents (C10), hypoglycemic agents (A10), and RAS-acting agents (C09). The prevalence of CV drug use was higher in women than in men for TCM products (9.07% vs. 5.74%, *p* < 0.05) and hypoglycemic agents (5.60% vs. 3.56%, *p* < 0.05). Of note, 67.78% participants did not receive any CV drugs, 13.67% took one type of CV drug, and 8.50% took at least two CV drugs (Table 2).

**TABLE 2:** Use of cardiovascular drugs in the total sample and by gender.

Cardiovascular drugs (ATC code)	Total sample (n = 5,246)	Men (n = 2,247)	Women (n = 2,999)	*p*-value^a^
Antithrombotic agents (B01)	364 (6.94)	161 (7.17)	203 (6.77)	0.576
Aspirin (B01AC06)	354 (6.75)	157 (6.99)	197 (6.57)	0.550
Clopidogrel (B01AC04)	10 (0.19)	4 (0.18)	6 (0.20)	0.856
Warfarin (B01AA03)	5 (0.10)	1 (0.04)	4 (0.13)	0.302
Diuretics (C03)	73 (1.39)	31 (1.38)	42 (1.40)	0.949
Beta-blockers (C07)	77 (1.47)	27 (1.20)	50 (1.67)	0.165
Calcium channel blockers (C08)	545 (10.39)	230 (10.24)	315 (10.50)	0.753
RAS-acting agents (C09)	242 (4.61)	99 (4.41)	143 (4.77)	0.536
Lipid-lowering agents (C10)	219 (4.17)	102 (4.54)	117 (3.90)	0.253
Hypoglycemic agents (A10)	248 (4.73)	80 (3.56)	168 (5.60)	0.001
Cardiac drugs (C01)	87 (1.66)	37 (1.65)	50 (1.67)	0.954
TCM products	401 (7.64)	129 (5.74)	272 (9.07)	<0.001
TCM for CVDs	323 (6.16)	112 (4.98)	211 (7.04)	0.002
TCM for hypertension	16 (0.30)	1 (0.04)	15 (0.50)	0.003
TCM for dyslipidemia	57 (1.09)	19 (0.85)	38 (1.27)	0.145
TCM for diabetes	31 (0.59)	3 (0.13)	28 (0.93)	<0.001
Numbers of cardiovascular drugs				<0.001
0	3,556 (67.78)	1,593 (70.89)	1963 (65.46)	
1	717 (13.67)	265 (11.79)	452 (15.07)	
2	446 (8.50)	190 (8.46)	256 (8.54)	
3	287 (5.47)	111 (4.94)	176 (5.87)	
4	151 (2.88)	57 (2.54)	94 (3.13)	
≥5	88 (1.68)	31 (1.38)	57 (1.90)	

Note: Data are n (%).

Abbreviations: ATC, Anatomical Therapeutic Chemical; CVD, cardiovascular disease; RAS, renin-angiotensin system; TCM, traditional Chinese medicine.

a p-value is for the test of differences between women and men.

### Cardiovascular Drug Use for Primary Prevention

Table 3 shows prevalence of CV drug use for primary prevention among participants who were free from CVD. The proportion of current use of CV drugs was 3.14% for antithrombotic agents (B01, mainly aspirin), 0.82% for diuretics (C03), 0.38% for beta-blockers (C07), 6.46% for calcium channel blockers (C08), 3.11% for RAS-acting agents (C09), 1.38% for lipid-lowering agents (C10), 3.70% for hypoglycemic agents (A10), 0.23% for cardiac drugs (C01), and 2.73% for TCM products (mostly for CVDs). The prevalence for use of CV drugs was higher in women than in men for TCM products (3.45% vs. 1.81%, *p* < 0.05) and hypoglycemic agents (4.40% vs. 2.81%, *p* < 0.05). There were no significant gender differences in use of any other CV drugs. Nearly 80% of the participants reported not taking any CV drug treatment.

**TABLE 3:** Use of cardiovascular drugs for primary prevention among participants free of cardiovascular disease.

Cardiovascular drugs (ATC code)	Total sample (n = 3,406)	Men (n = 1,495)	Women (n = 1911)	*p*-value^a^
Antithrombotic agents (B01)	107 (3.14)	49 (3.28)	58 (3.04)	0.687
Aspirin (B01AC06)	105 (3.08)	47 (3.14)	58 (3.04)	0.855
Clopidogrel (B01AC04)	2 (0.06)	2 (0.13)	0	0.110
Warfarin (B01AA03)	1 (0.03)	0	1 (0.05)	0.376
Diuretics (C03)	28 (0.82)	12 (0.80)	16 (0.84)	0.912
Beta-blockers (C07)	13 (0.38)	7 (0.47)	6 (0.31)	0.469
Calcium channel blockers (C08)	220 (6.46)	89 (5.95)	131 (6.86)	0.288
RAS-acting agents (C09)	106 (3.11)	42 (2.81)	64 (3.35)	0.368
Lipid-lowering agents (C10)	47 (1.38)	22 (1.47)	25 (1.31)	0.685
Hypoglycemic agents (A10)	126 (3.70)	42 (2.81)	84 (4.40)	0.015
Cardiac drugs (C01)	8 (0.23)	4 (0.27)	4 (0.21)	0.728
TCM products	93 (2.73)	27 (1.81)	66 (3.45)	0.003
TCM for CVDs	56 (1.64)	19 (1.27)	37 (1.94)	0.130
TCM for hypertension	5 (0.15)	0	5 (0.26)	0.048
TCM for dyslipidemia	23 (0.68)	6 (0.40)	17 (0.89)	0.084
TCM for diabetes	20 (0.59)	2 (0.13)	18 (0.94)	0.002
Numbers of cardiovascular drugs				0.121
0	2,600 (76.34)	1,171 (78.33)	1,429 (74.78)	
1	437 (12.83)	177 (11.84)	260 (13.61)	
2	233 (6.84)	98 (6.56)	135 (7.06)	
3	92 (2.70)	33 (2.21)	59 (3.09)	
4	30 (0.88)	9 (0.60)	21 (1.10)	
≥5	14 (0.41)	7 (0.47)	7 (0.37)	

Note: Data are n (%).

Abbreviations: ATC, Anatomical Therapeutic Chemical; CVD, cardiovascular disease; RAS, renin-angiotensin system; TCM, traditional Chinese medicine.

a p-value is for the test of differences between women and men.

### Cardiovascular Drug Use for Secondary Prevention

Table 4 shows rates of CV drug use in participants with CVD for the secondary prevention. Among all CV drugs, the proportion of current use of CV drugs was 13.97% for antithrombotic agents (B01, mainly aspirin), 2.45% for diuretics (C03), 3.48% for beta-blockers (C07), 17.66% for calcium channel blockers (C08), 7.39% for RAS-acting agents (C09), 9.35% for lipid-lowering agents (C10), 6.63% for hypoglycemic agents (A10), 4.29% for cardiac drugs (C01), and 16.74% for TCM products. Over 50% of participants with CVDs reported not taking any CV drug treatment, whereas nearly 17% of them concurrently used three or more of the CV drugs.

**TABLE 4:** Use of cardiovascular drugs for secondary prevention among participants with cardiovascular disease.

Cardiovascular drugs (ATC code)	Total sample (n = 1840)	Men (n = 752)	Women (n = 1,088)	*p*-value^a^
Antithrombotic agents (B01)	257 (13.97)	112 (14.89)	145 (13.33)	0.341
Aspirin (B01AC06)	249 (13.53)	110 (14.63)	139 (12.78)	0.254
Clopidogrel (B01AC04)	8 (0.43)	2 (0.27)	6 (0.55)	0.360
Warfarin (B01AA03)	4 (0.22)	1 (0.13)	3 (0.28)	0.518
Diuretics (C03)	45 (2.45)	19 (2.53)	26 (2.39)	0.852
Beta-blockers (C07)	64 (3.48)	20 (2.66)	44 (4.04)	0.111
Calcium channel blockers (C08)	325 (17.66)	141 (18.75)	184 (16.91)	0.309
RAS-acting agents (C09)	136 (7.39)	57 (7.58)	79 (7.26)	0.797
Lipid-lowering agents (C10)	172 (9.35)	80 (10.64)	92 (8.46)	0.114
Hypoglycemic agents (A10)	122 (6.63)	38 (5.05)	84 (7.72)	0.024
Cardiac drugs (C01)	79 (4.29)	33 (4.39)	46 (4.23)	0.868
TCM products	308 (16.74)	102 (13.56)	206 (18.93)	0.002
TCM for CVDs	267 (14.51)	93 (12.37)	174 (15.99)	0.030
TCM for hypertension	11 (0.60)	1 (0.13)	10 (0.92)	0.032
TCM for dyslipidemia	34 (1.85)	13 (1.73)	21 (1.93)	0.753
TCM for diabetes	11 (0.60)	1 (0.13)	10 (0.92)	0.032
Numbers of cardiovascular drugs				0.004
0	956 (51.96)	422 (56.12)	534 (49.08)	
1	280 (15.22)	88 (11.70)	192 (17.65)	
2	213 (11.58)	92 (12.23)	121 (11.12)	
3	195 (10.60)	78 (10.37)	117 (10.75)	
4	122 (2.63)	48 (6.38)	74 (6.80)	
≥5	74 (4.02)	24 (3.19)	50 (4.60)	

Note: Data are n (%).

Abbreviations: ATC, Anatomical Therapeutic Chemical; CVD, cardiovascular disease; RAS, renin-angiotensin system; TCM, traditional Chinese medicine.

a p-value is for the test of differences between women and men.

Table 5 shows the use of various classes of CV drugs in patients with IHD, HF, AF, or stroke. Patients with stroke were more likely to use antithrombotic agents (B01, 17.62%), calcium channel blockers (C08, 22.26%), and lipid-lowering agents (C10, 12.02%), compared with patients with IHD (13.72%, 16.58%, and 9.46%, respectively). The use of beta-blockers (C07), RAS-acting agents (C09), diuretics (C03), and cardiac drugs (C01) in patients with HF was 3.92%, 7.84%, 3.92%, and 8.50%, respectively. The overall utilization of antithrombotic agents (B01) in patients with AF was 14.29%, and the use of warfarin (B01AA03) was only 2.38%. Moreover, ∼50% of people with IHD, AF, or stroke, and ∼45% of people with HF did not take any CV drugs.

**TABLE 5:** Use of cardiovascular drugs by cardiovascular disease.

Cardiovascular drugs (ATC code)	Ischemic heart disease (n = 1,152)	Heart failure (n = 153)	Atrial fibrillation (n = 84)	Stroke (n = 840)
Antithrombotic agents (B01)	158 (13.72)	24 (15.69)	12 (14.29)	148 (17.62)
Aspirin (B01AC06)	154 (13.37)	24 (15.69)	10 (11.90)	143 (17.02)
Clopidogrel (B01AC04)	6 (0.52)	1 (0.65)	1 (1.19)	5 (0.60)
Warfarin (B01AA03)	2 (0.17)	0	2 (2.38)	2 (0.24)
Diuretics (C03)	32 (2.78)	6 (3.92)	4 (4.76)	19 (2.26)
Beta-blockers (C07)	54 (4.69)	6 (3.92)	6 (7.14)	24 (2.86)
Calcium channel blockers (C08)	191 (16.58)	25 (16.34)	11 (13.10)	187 (22.26)
RAS-acting agents (C09)	91 (7.90)	12 (7.84)	7 (8.33)	67 (7.98)
Lipid-lowering agents (C10)	109 (9.46)	15 (9.80)	7 (8.33)	101 (12.02)
Hypoglycemic agents (A10)	80 (6.94)	13 (8.50)	4 (4.76)	58 (6.90)
Cardiac drugs (C01)	70 (6.08)	13 (8.50)	14 (16.67)	25 (2.98)
TCM products	202 (17.53)	44 (28.76)	12 (14.29)	147 (17.50)
TCM for CVDs	179 (15.54)	38 (24.84)	10 (11.90)	125 (14.88)
TCM for hypertension	6 (0.52)	2 (1.31)	0	3 (0.36)
TCM for dyslipidemia	19 (1.65)	4 (2.61)	2 (2.38)	22 (2.62)
TCM for diabetes	8 (0.69)	2 (1.31)	0	4 (0.48)
Number of cardiovascular drugs				
0	578 (50.17)	69 (45.10)	43 (51.19)	425 (50.60)
1	198 (17.19)	20 (13.07)	14 (16.67)	107 (12.74)
2	121 (10.50)	26 (16.99)	9 (10.71)	95 (11.31)
3	121 (10.50)	20 (13.07)	4 (4.76)	101 (12.02)
4	79 (6.86)	7 (4.58)	11 (13.10)	73 (8.69)
≥5	55 (4.77)	11 (7.19)	3 (3.57)	39 (4.64)

Note: Data are n (%).

Abbreviations: ATC, Anatomical Therapeutic Chemical; CVD, cardiovascular disease; RAS, renin-angiotensin system; TCM, traditional Chinese medicine.

## Discussion

Major findings from this large-scale community-based study of rural-dwelling older adults can be summarized into two points. First, overall, a very low proportion of older adults in the rural settings in China reported taking CV medications for primary and secondary prevention of CVDs, despite highly prevalent cardiovascular risk factors and CVDs; Second, this study investigated the use of TCM products for primary and secondary CVD prevention. TCM products were the second most commonly used drugs in the total sample, and the prevalence for use of TCMs was higher in women than in men. These findings have relevant policy implications for improving preventive and therapeutic management of CVDs among older adults living in the rural communities in China.

The management of long-term chronic health conditions (e.g., CVDs) is increasingly shifting from secondary care to general practitioners. The decreasing trends in CVD mortality since the 1980s in the United Kingdom are thought partly to be due to better early treatment (Townsend et al., 2015; Bhatnagar et al., 2016). Data on medicine use from two comparable population-based studies of older people (age 65 + years) in England showed that the proportion of people who did not take any medication had decreased from around one-fifth to one-thirteenth during the past 2 decades (Gao et al., 2018). Data from the Swedish National study on Aging and Care in Kungsholmen (SNAC-K) showed that antithrombotic agents and diuretics were the most commonly prescribed medications among elderly people aged ≥60 years, with over 20% of older adults using these drugs (Ding et al., 2014). However, underuse of CV medications and poor control of risk factors are still very serious in the rural settings and low- and middle-income countries (Yusuf et al., 2011). In our study, two-thirds of the participants did not receive any CV drugs. The two most commonly used CV drugs, calcium channel blockers and TCM products, were reported to be used in less than 10% of older adults. The suboptimal use of simple, inexpensive preventive CV drug therapies suggests that implementation of the proven strategies to reduce the risk and the burden of CVDs remains to be strengthened in the rural areas in China.

The prevalence of cardiovascular risk factors and CVDs increases as people age and pharmacological therapies are crucial to reduce risk of cardiovascular events and mortality (Karmali et al., 2016). Statin therapy for people with dyslipidemia is recommended as the first-line treatment for primary prevention of CVDs (Arnett et al., 2019), which could reduce the risk of major cardiovascular events by 21% (Baigent et al., 2005). The total prevalence of dyslipidemia in our study population was above 20%. However, only 4.17% of the participants received lipid-lowering drug treatment. Hypertension is common among older adults, yet it remains inadequately controlled (Lewington et al., 2016). Our results are consistent with a previous study (Lu et al., 2017) that hypertension affected two-thirds of the study participants, but fewer than a third were being treated with antihypertensive drugs, and calcium channel blockers was the most commonly used class of antihypertensive medications. This suggests that pharmacological management of certain cardiovascular risk factors among the community-dwelling older adults needs to be improved.

It has been well established that antithrombotic treatment has beneficial effects for secondary prevention of coronary heart disease, stroke, and AF (January et al., 2019). In our study, antithrombotic agents were used by only 14.29% of people with AF, which appears to be inadequate compared to the reports from a previous hospital-based study of patients with AF (Yu et al., 2012). Furthermore, despite the preventive effect of warfarin against stroke is superior to antiplatelet agents among patients with AF, the underuse of warfarin in clinical practice has been reported in several studies (Garcia and Hylek, 2006; Stramba-Badiale, 2008). Use of RAS-acting agents, such as ACE inhibitors, has been considered to be cost-effective with regard to reduction of mortality and hospitalization in older patients with HF (Weintraub et al., 2002). However, in our study only 7.84% of patients with HF used RAS-acting agents, which is substantial below the optimal level (Klarin et al., 2003; Rushton et al., 2014). Moreover, evidence has shown that combination pharmacotherapy for secondary prevention of CVDs could reduce the overall risk of all-cause mortality by approximately 40% and cardiovascular events by 25%–30% compared to either monotherapy or no therapy (Aalto-Setala et al., 2019). Nevertheless, less than 30% of the participants in our study used two or more CV drugs together (including TCM products). This suggests that additional efforts are imperative to promote implementation of the evidence-based recommendations for secondary prevention of CVDs in the primary care settings in the rural regions.

Current evidence indicates that some TCM medications might be effective in control of cardiovascular risk factors and might be used as a complementary and alternative approach to the primary and secondary prevention of CVDs such as IHD and HF (Hao et al., 2017). The primary healthcare system in China includes a routine TCM health check and education for residents aged 65 years or older (Li et al., 2017). Our data showed that 7.64% of all participants took TCM products, and 16.74% of participants with CVD took TCM products. The most commonly used TCM product was Salvia miltiorrhiza Bunge (34.91%), which is a kind of herbs in formulations frequently prescribed for the clinical treatment of CVD in China (Wang et al., 2017). The active compounds of Salvia miltiorrhiza Bunge are considered to have cardioprotective effects through different cell signaling pathways (Ho and Hong, 2011). Thus, these TCM products might play a role in primary and secondary prevention of CVDs. However, TCM products have not been recommended in the current national guidelines of CVD management. Additional evidence from high-quality research (e.g., randomized controlled trials) is needed to support the effectiveness of TCM products in the prevention and treatment of CVDs (Gang et al., 2017).

Our findings corroborate and extend previous reports by showing that residents in the rural settings of China are less likely to receive CV drugs. Several factors could affect the use of CV drug therapy for primary and secondary prevention of CVDs, such as low awareness of cardiovascular risks and self-perceived health consequences, the failure of timely detection and diagnosis of risk factors and CVDs, concerns about adverse effects of CV drugs, and affordability (Haynes et al., 2008; Niens et al., 2010). Indeed, health insurance coverage could partly explain some of the disparities in CVD prevention. Although the majority of the rural population (∼95%) have joined the New Rural Cooperative Medical Scheme, it only covers costs for in-hospital care and treatment, whereas long-term therapies with antihypertensive medications, statins, and antiplatelet agents still require out-of-pocket payment (Gu et al., 2015). Additionally, the prevalence of current harmful alcohol drinking and smoking in our rural population was above one-third and one-fifth, respectively. This might influence CV drug treatment for CVD prevention because regular smokers or alcohol drinkers may reflect the so-called “crowding out effect” where the costs of smoking and alcohol drinking compromise the allocation of expenditure for essential health care and treatment (Zhang et al., 2018).

The major strength of our study refers to the large sample of the general elderly population from the rural settings in China. In addition, our assessments with regard to risk factors and medical history were performed by trained staff following the standardized procedures. Of note, our study also covered the use of TCM products for control of cardiovascular risk factors and CVDs, which is unique and has been rarely reported in previous studies. However, our study also has limitations. First, despite our efforts to diagnose various CVDs through face-to-face survey on medical history, clinical and neurological examinations, and ECG examination, some CVDs might still be missed. Second, we did not take into account adherence to the reported treatments and outcome measures. Third, the study participants were derived exclusively from the rural areas with limited education and low-to-medium socioeconomic status, so cautiousness is needed when generalizing our research findings to other populations, even other rural populations.

In conclusion, health care reform in rural China since 2009 has significantly improved the rural public healthcare system by meeting the need of health care of rural residents (Baradaran et al., 2013). However, there is still a gap in the preventive and therapeutic management of CVDs with regard to the evidence-based recommendations for primary and secondary prevention in primary care. Furthermore, despite the fact that some TCM products might have potential preventive and therapeutic effects on cardiovascular risk factors and related CVDs, rigorously designed randomized controlled trials are warranted to evaluate the cardiovascular benefits of TCM products for the primary and secondary prevention of CVDs.

## Data Availability Statement

The raw data supporting the conclusions of this article will be made available by the authors, without undue reservation.

## Ethics Statement

The MIND-China protocol has been approved by the Ethics Committee at Shandong Provincial Hospital, Jinan, Shandong, China. Written informed consent was obtained from all participants, or in the case of cognitively impaired persons, from a proxy (usually a guardian or a family member).

## Author Contributions

The study concept and design: LC, YW, YD, and CQ. Writing of the manuscript: LC. Data analysis: YR and LC. Obtaining data: LC, YW, YR, TH, XH, YD, QZ, RL, LW, and SX. Interpretation of results, critical revisions of the manuscript, and approval of the version for submission: All authors.

## Funding

This work was supported in part by grants from the National Key R&D Program of China (grant no.: 2017YFC1310100), the National Natural Science Foundation of China (grants no.: 81861138008), the Academic Promotion Program of Shandong First Medical University, and the Taishan Scholar Program of Shandong Province, China. CQ received grants from the Swedish Research Council (grants no.: 2017-00740 and 2017-05819) for the Sino-Sweden Network and Joint Research Projects and the Swedish Foundation for International Cooperation in Research and Higher Education (STINT, grant no.: CH 2019-8320) for the Joint China-Sweden Mobility program, Stockholm, Sweden. The funding agency had no role in the study design, data collection and analysis, writing of this manuscript, and in the decision to submit the work for publication.

## Conflict of Interest

The authors declare that the research was conducted in the absence of any commercial or financial relationships that could be construed as a potential conflict of interest.
